# Morphological Characterization and Genetic Diversity of Rice Blast Fungus, *Pyricularia oryzae*, from Thailand Using ISSR and SRAP Markers

**DOI:** 10.3390/jof6010038

**Published:** 2020-03-19

**Authors:** Apinya Longya, Sucheela Talumphai, Chatchawan Jantasuriyarat

**Affiliations:** 1 Department of Genetics, Faculty of Science, Kasetsart University, Bangkok 10900, Thailand; apinya.longya@gmail.com; 2 Major Biology, Department of Science and Technology, Faculty of Liberal Arts and Science Roi Et Rajabhat University, Roi Et 45120, Thailand; aor_sucheela@hotmail.com; 3 Center for Advanced Studies in Tropical Natural Resources, National Research University-Kasetsart University (CASTNAR, NRU-KU), Kasetsart University, Bangkok 10900, Thailand

**Keywords:** ascomycete, genetic diversity, ISSR marker, *Pyricularia oryzae*, rice blast, SRAP marker

## Abstract

Rice blast disease is caused by the ascomycete fungus *Pyricularia oryzae* and is one of the most destructive rice diseases in the world. The objectives of this study were investigating various fungal morphological characteristics and performing a phylogenetic analysis. Inter-simple sequence repeat (ISSR) and sequence-related amplified polymorphism (SRAP) markers were used to examine the genetic variation of 59 rice blast fungus strains, including 57 strains collected from different fields in Thailand and two reference strains, 70-15 and Guy11. All isolates used in this study were determined to be *P. oryzae* by internal transcribed spacer (ITS) sequence confirmation. A total of 14 ISSR primers and 17 pairs of SRAP primers, which produced clear and polymorphic bands, were selected for assessing genetic diversity. A total of 123 polymorphic bands were generated. The similarity index value for the strains ranged from 0.25 to 0.95. The results showed that the blast fungus population in Thailand has both morphological and genetic variations. A high level of genetic variation, or genome adaptation, is one of the fungal mechanisms that could overcome host resistance to avoid host recognition. Results from this research study could bring substantial benefits and ultimately help to understand the blast fungal pathogen genome and the population structure in Thai blast fungus.

## 1. Introduction

The ascomycete fungus *Pyricularia oryzae* (teleomorph: *Magnaporthe oryzae*), is one of the most damaging rice diseases in the world and is able to cause a yield loss of up to 30% and infect more than 50 species of grasses, including wheat, barley, oat, and millet [1]. Resistant rice cultivars have been used to control blast disease, but they usually lose resistance within a few years of release because of fungal genome adaptation that escape plant immunity [2]. The genetic diversity of the pathogen has limited the effectiveness of this approach. Understanding the structure of the blast population and genetic diversity are important for managing the disease. 

DNA profiling techniques that have been successfully used to assess genetic diversity include inter-simple sequence repeat (ISSR), random-amplified polymorphic DNA (RAPD), and amplified fragment length polymorphism (AFLP) [3]. They are dominant markers, which use universal primers for anonymous regions to extensively analyze the genetic diversity [4]. More recently, sequence-related amplified polymorphism (SRAP) markers have been developed, which are used to amplify coding regions with primers targeting open reading frames (ORFs) [5]. The molecular markers differ in cost, speed, complication, labor, degree of polymorphism, and repeatability. ISSR and SRAP marker techniques are fast and low cost, they do not require sequence information, have high repeatability, and use only one-step PCR. They provide highly discriminating information with good reproducibility and are relatively abundant, while AFLP and RAPD are more labor intensive and time consuming [4,6]. We characterized blast fungal morphology, performed DNA sequence analyses of the internal transcribed spacer (ITS), and used 20 ISSR and 30 SRAP markers to assess the genetic diversity of 59 rice blast strains, including 57 strains collected from Thai rice-growing areas and two reference strains, 70-15 and Guy11. The information obtained from this study will help to understand the population structure and evolution of rice blast fungus in Thailand.

## 2. Materials and Methods 

### 2.1. Rice Blast Materials 

A total of 59 rice blast strains were used, including 57 strains collected from Thai rice-growing areas representing central, northern, and north-eastern parts of Thailand. Two rice blast strains, 70-15 and Guy11, were used as reference strains (Table 1). The stock of each blast strain as in filter paper was cultured and DNA was extracted using the cetyl-trimethyl-ammonium-bromide (CTAB) method described by Longya et al. [7]. 

### 2.2. Fungal Morphology Characterization 

Each blast fungal strains as in filter paper was cultured on rice flour agar (RFA) at 28 °C for 5 days, then a cork borer was used to cut a hole of the activated mycelium and was transferred to a new RFA plate, followed by incubation at 28 °C. Mycelium radii were measured at 4, 6, 8, and 10 days after incubation and each series was repeated 3 times. Mycelial characteristics on agar plates, including appearance, color, and amount of melanin pigment produced, were recorded. Conidia production was stimulated by scraping the surface of the fungal mycelium with a sterile spreader and incubating under ultraviolet light (black light) for 2 days. Conidia were harvested with sterile water.

### 2.3. ITS Amplification and Sequencing

ITS regions (18S rDNA, ITS1, 5.8S rDNA, and ITS2) were amplified using the universal primers ITS5 (forward primer; 5′-GGAAGTAAAAGTCGTAACAAGG-3′) and ITS4 (reverse primer; 5′-TCCTCCGCTTATTGATATGC-3′). Polymerase chain reaction (PCR) amplification was conducted in a 25 µL reaction volume containing 100 ng of DNA template, 2.5 µL of 10× PCR buffer (200 mM Tris-HCl, 500 mM KCl), 2 mM of MgCl_2_, 0.2 mM of each dNTP, 0.5 µM of each primer, and 1 unit of Taq DNA polymerase (Vivantis, Shah Alam, Malaysia). PCR was performed using a Mastercycler nexus (Eppendorf, Hamburg, Germany) under the following conditions: Initial denaturation at 95 °C for 5 min; 35 cycles of denaturation at 95 °C for 30 s, annealing at 55 °C for 30 s, and elongation at 72 °C for 1 min, as well as a final extension at 72 °C for 5 min. The amplification products were resolved in 1% agarose gel using 0.5× TBE buffer at 100 V for 45 min followed by DNA staining with EtBr and analysis using a Gel Doc XR+ with Image Lab software (Bio-Rad, Hercules, CA USA). PCR products were purified using the GF-1 PCR Clean-Up Kit (Vivantis, Shah Alam, Malaysia) based on the manufacturer’s instructions. The purified PCR products were sequenced with the ITS4 primer by a commercial sequencing service provider (U2Bio, Bangkok, Thailand). The resulting sequences of each isolate were refined using a BioEdit sequence alignment editor. To analyze relationships between isolates, all 59 sequences from this study and sequences of 10 reference *Pyricularia oryzae* isolates from difference hosts (obtained from the GenBank) were aligned using the MEGA X (Version 10.1.7) with *Pyricularia pennisetigena* (MN947526) used as an outgroup. 

### 2.4. ISSR Analysis

A total of 20 ISSR primers (Table 2) were used for PCR. PCR reactions were performed in a total volume of 25 μL containing 1× PCR buffer, 0.25 mM dNTPs, 5 U Taq polymerase (Vivantis, Shah Alam, Malaysia), 0.8 μmol ISSR primer, and 100 ng DNA template. PCR was performed using a Mastercycler nexus (Eppendorf, Hamburg, Germany) under the following conditions: Initial activation at 94 °C for 5 min; 35 cycles of denaturation at 94 °C for 1 min, annealing at 55 °C for 1 min, and extension at 72 °C for 2 min, as well as final incubation at 72 °C for 10 min. PCR products were visualized by separation in a 1.5% agarose gel followed by DNA staining with EtBr and analysis using a Gel Doc XR+ with Image Lab software (Bio-Rad, Hercules, CA USA). All primers were repeated at least twice.

### 2.5. SRAP Analysis

A total of 5 forward and 6 reverse primers of SRAP markers (for a total of 30 primer combinations) were used (Table 3). The PCR reaction was in a total volume of 25 μl containing 1× PCR buffer, 0.5 mM dNTPs, 1.5 U Taq polymerase (Vivantis, Shah Alam, Malaysia), 0.2 μmol forward primer and reverse primer, and 50 ng DNA template. PCR was performed using a Mastercycler nexus (Eppendorf, Hamburg, Germany) under the following conditions: Initial activation at 94 °C for 5 min; 6 cycles of denaturation at 94 °C for 1 min, annealing at 35 °C for 40 s, and extension at 72 °C for 2 min; 32 cycles of denaturation at 94 °C for 1 min, annealing at 46 °C for 40 s, and extension at 72 °C for 2 min; and final incubation at 72 °C for 10 min. PCR products were visualized by separation in a 1.5% agarose gel followed by DNA staining with EtBr and analysis using a Gel Doc XR+ with Image Lab software (Bio-Rad, Hercules, CA USA). All primers were repeated at least twice.

### 2.6. Data Analysis

Amplification products were scored as present (1), absent (0), or missing data (9). The level of polymorphism for each primer was represented by the percentage of polymorphic variable loci relative to all the loci analyzed. Dendrograms were constructed using the unweighted pair-group method with arithmetic means (UPGMA) for clustering. For each of the dendrograms obtained from the ISSR, SRAP, and ISSR+SRAP combination data, a cophenetic matrix was generated using NTSYSpc version 2.01. The polymorphism information content (PIC) of an individual locus for dominant markers (ISSR and SRAP markers) was calculated as:

2ƒi(1–ƒi),
(1)

where *f*i indicates the frequency of bands presence for locus *i* [8].

## 3. Results

### 3.1. Fungal Morphology Variation

**Figure 1:**
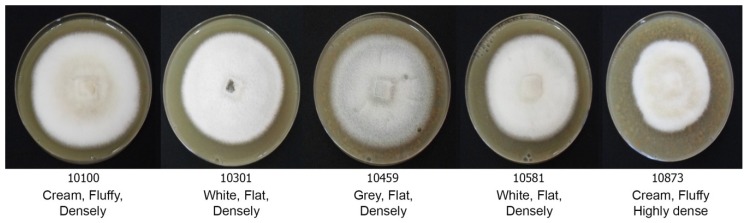
Variation in morphology of *Pyricularia oryzae* mycelia on the rice flour agar (RFA) medium, showing differences in colors and mycelial growth patterns.

**Figure 2:**
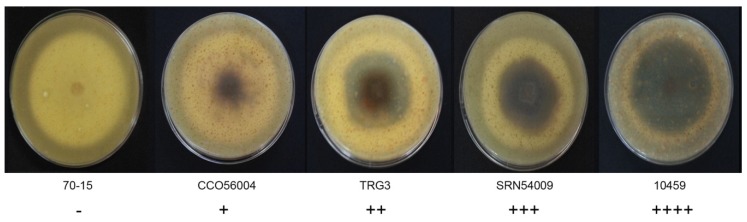
Variation in *Pyricularia oryzae* melanin production on ventral view of the rice flour agar (RFA) medium, showing differences in melanin production ability. Scoring: No melanin pigment (-), melanin pigment production in less than 20% of the mycelia area (+), melanin production in 20%–50% of the mycelia area (++), melanin production in 50%–80% of the mycelia area (+++), and melanin production in more than 80% of the mycelia area (++++).

**Figure 3:**
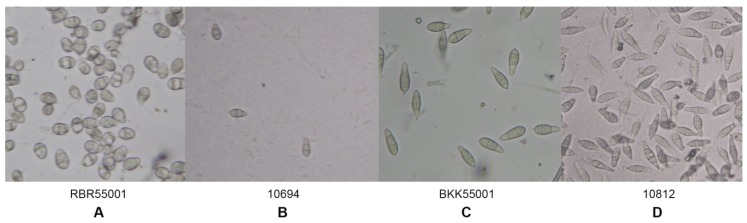
Variation in conidia morphology shape under a light microscope. A: Pattern A, oval conidia; B: Pattern B, short pyriform conidia; C: Pattern C, pyriform conidia; and D: Pattern D, long pyriform conidia.

The morphological characteristics of 59 rice blast strains were examined. Fungal filaments on RFA plates were observed, and each strain showed different characteristics in terms of appearance and color. Three colors were recorded: White, cream, and grey. Flat or fluffy filaments and filament density were observed for filamentous pattern (Table 1 and Figure 1). After 10 days of culture, melanin pigment was observed from a ventral view of the RFA plate and each strain was classified based on their melanin production capability and given one of five scores, - to ++++, depending on the amount of melanin they produced (Table 1 and Figure 2). Fungal growth was measured by the mycelial radius at four, six, eight, and 10 days after incubation (Supplemental Data S1). The average radius of fungal mycelium at four, six, eight, and 10 days were 8.70, 15.36, 21.78, and 26.94 mm with standard deviations of 0.6, 1.13, 1.76, and 2.34 mm, respectively. The conidia shape was observed under a microscope and was classified as one of four patterns: Oval shape (pattern A, Figure 3A), short pyriform shape (pattern B, Figure 3B), pyriform shape (pattern C, Figure 3C), and long pyriform shape (pattern D, Figure 3D). Most conidia were pyriform shape (77.97%, 46/59). Four of 59 (6.78%) of the blast fungal isolates had short pyriform conidia, eight isolates (13.60%) were long pyriform conidia, and one strain (RBR55001) (1.69%) produced oval shape conidia (Table 1).

### 3.2. ITS Sequence Analysis

**Figure 4:**
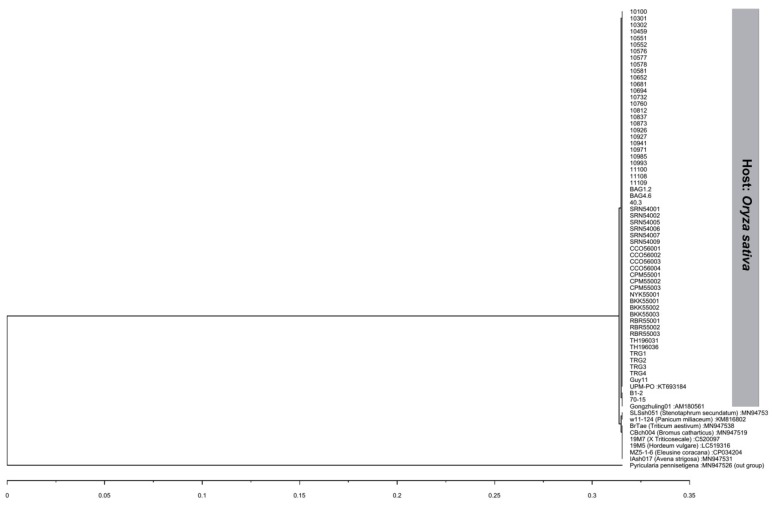
Dendrogram based on the internal transcribed spacer (ITS) of the 59 isolates used in this study compared to the reference *Pyricularia oryzae* sequences from different hosts obtained from GenBank based on UPGMA cluster analysis. Hosts are specified in parentheses, and GenBank accession numbers are provided following the colon.

The ITS gene regions were successfully amplified and were 567 bp long, of which 497 bp contained an 18S ribosomal RNA partial sequence and complete sequences of internal transcribed spacer 1, the 5.8S ribosomal RNA gene, and internal transcribed spacer 2. BLASTn queries indicated that all isolates used in this study were 100% identical to *P. oryzae* in GenBank. 57 of 59 isolates had the same nucleotide sequence as the rice blast reference isolate UPM-PO (accession no. KT693184). Two isolates, B1-2 and 70-15, had a 99.80% identity with most isolates with one nucleotide substitution at position 403 (T/G), which is located in the ITS2 region. However, both isolates were 100% identical with another rice blast reference isolate, Gongzhuling01 (accession no. AM180561). All isolates were deposited in GenBank with accession numbers MT126183–MT126241 (Table 1). Interestingly, isolates B1-2 and 70-15 had mating type locus MAT1-1, while the rest of the isolates had mating type locus MAT1-2. A dendrogram depicted by UPGMA analysis was constructed using the Thai rice blast isolates with eight *P. oryzae* from difference hosts. The dendrogram showed that all Thai rice blast isolates were in the same clade with the reference rice blast isolate UPM-PO (accession no. KT693184) and isolate Gongzhuling01 (accession no. AM180561) and were separated from blast isolates from other host species. The isolate SLSsh051 (host: *Stenotaphrum secundatum*) and isolate w11-124 (host: *Panicum miliaceum*) were in the same clade (Figure 4), and another clade comprise blast pathogens from six different hosts: Isolates BrTae (host: *Triticum aestivum*), CBch004 (host: *Bromus catharticus*), 19M7 (host: *x Triticosecale*), 19M5 (host: *Hordeum vulgare*), MZ5-1-6 (host: *Eleusine coracana*), and IAsh017 (host: *Avena strigosa*). The results confirmed that all isolates used in this study belonged to the *P. oryzae* rice blast pathogen.

### 3.3. Genetic Polymorphism from ISSR Marker

**Figure 5:**
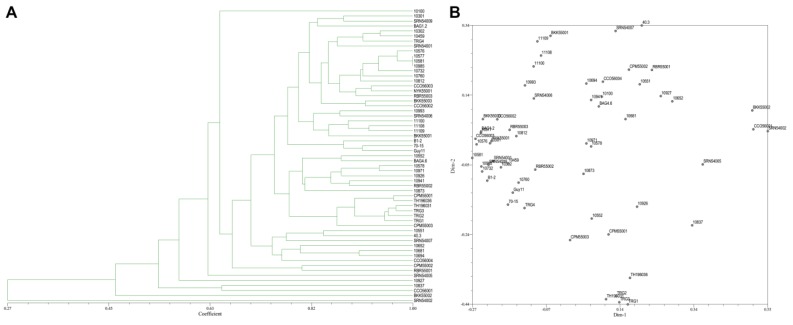
(**A**), Dendrogram presenting the relationship from ISSR markers based on unweighted pair-group method with arithmetic means (UPGMA) cluster analysis. (**B**), 2D principal component analysis (PCA) based on genetic relationships from ISSR data using the DECENTER and EIGEN features of NTSYSpc.

From the 20 ISSR primers, 14 primers showed reproducible and polymorphic DNA amplification patterns. 118 DNA bands were obtained with an average of 8.43 DNA bands per primer, and 62 DNA bands (52.90%) were polymorphic bands with an average of 4.43 polymorphic DNA bands per primer (Table 2). The vast majority of ISSR primers showed a moderate level of polymorphism. PIC scores of each primer ranged from 0.07 (ISSR18) to 0.50 (ISSR14, ISSR15, and ISSR17), with an average of 0.36. A genetic similarity ranged from 0.27–1.00. A dendrogram and two-dimensional principal component analysis (PCA) presenting the relationship from ISSR markers based on UPGMA cluster analysis showed that the rice blast population in Thailand is a diverse, sparse arrangement that does not classify into a group (Figure 5A,B).

### 3.4. Genetic Polymorphism from SRAP Marker

**Figure 6:**
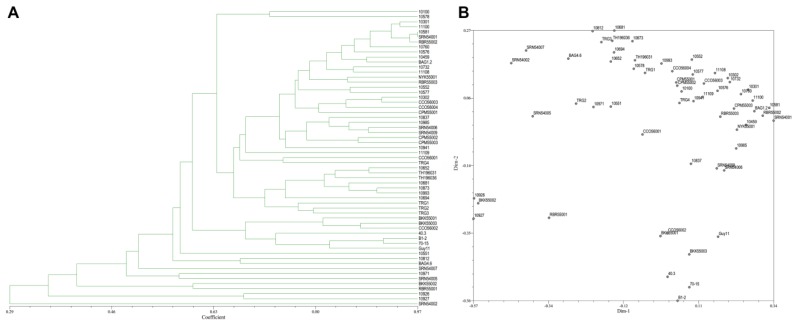
(**A**), Dendrogram presenting the relationship from SRAP based on UPGMA cluster analysis. (**B**), 2D principal component analysis (PCA) based on genetic relationships from SRAP data using the DECENTER and EIGEN features of NTSYSpc.

From the 30 SRAP primer combinations, 17 combinations showed clear reproducible band patterns and exhibited polymorphism. The results showed 103 DNA bands with 61 polymorphic bands (68.22%). The average number of generated DNA bands and polymorphic DNA bands per primer were 5.72 and 3.38, respectively (Table 3). The vast majority of SRAP primers showed a moderate level of polymorphism. PIC scores of each primer ranged from 0.03 (SRAP29) to 0.50 (SRAP1, SRAP6, SRAP8, and SRAP24), with an average of 0.39. The coefficients of similarity ranged from 0.29–0.97. A dendrogram and two-dimensional PCA presenting the relationship from SRAP markers based on UPGMA cluster analysis showed that the rice blast population in Thailand is diverse (Figure 6A,B).

### 3.5. Genetic Polymorphism from ISSR and SRAP Markers Combination

**Figure 7:**
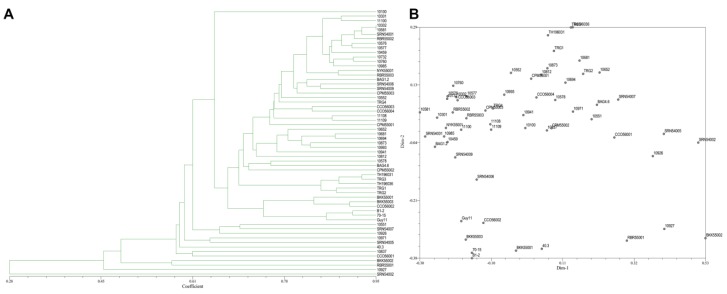
(**A**), Dendrogram presenting the relationship from ISSR markers and SRAP markers based on UPGMA cluster analysis. (**B**), 2D principal component analysis (PCA) based on genetic relationships from ISSR and SRAP data using the DECENTER and EIGEN features of NTSYSpc.

A total of 123 polymorphic DNA bands from the combined data of ISSR and SRAP markers were used to construct a dendrogram. The coefficients of similarity ranged from 0.28–0.95. The dendrogram and two-dimensional PCA presenting the relationship from ISSR and SRAP markers based on UPGMA cluster analysis showed that most rice blast strains were clustered together with only a few strains separated from the main group (Figure 7A,B).

## 4. Discussion

### 4.1. Morphological Characteristics of Rice Blast Fungus Strains in Thailand are Diverse

Studies on the morphological characteristics of different rice blast fungus strains in Thailand revealed variations with respect to mycelium color, morphology, and conidia shape. Colony color varied from white to grey. Some strains were unable to produce melanin pigment, while most showed a range of production capabilities. The colony radii of different isolates were recorded, and the standard deviations at day four, six, eight, and 10 were 0.6, 1.13, 1.76, and 2.34 mm, respectively. At the beginning, all strains grew at similar rates, but they eventually began growing at different rates and with unique patterns. This led to the production of different mycelium morphologies. Isolate 10873, due to its highly dense mycelium, showed slower mycelium growth when compared with the others, which is a similar result to a previous report by Sonah et al. [9] in that both color and mycelium grown pattern varied greatly with strains on oat meal agar. The Magnaporthaceae are distinguished from the Pyriculariaceae by their asexual morphs, which are Phialophora- or Harpophora-like. Additionally, Magnaporthaceae have falcate, versicolored conidia on brown, erect conidiophores, while Pyricularia or Pyricularia-like species are characterized by pyriform two-septate conidia, three-celled with difference. The size and shape of spores are an important criteria for the classification and identification of *Pyricularia* species [9]. Strains significantly varied in spore morphology depending on specific hosts. Some isolates derived from non-rice hosts also showed abnormal spore morphology as spores were longer, cylindrical, and obpyriform [9]. In our results, we found varied conidial shapes, from oval to long pyriform. Most conidia were a pyriform shape (46/59). Only one strain (RBR55001) produced oval conidia and this was different from other strains as well as from previous reports. Many epidemiological studies on the relationship between the formations, dispersal, and infection behavior of spores and environmental factors have been reported [10]. The fungal growth and its morphology are affected by different environmental factors, such as temperature, pH, moisture, degree of aeration, and amount and type of nutrients [11]. Furthermore, mycovirus infection in *P. oryzae* causes morphological changes, including changes in their growth on agar, melanin biosynthesis, and formation of conidia and appressoria [12]. 

Due to the overlap of some morphological characteristics and host-specific confirmation, ITS DNA sequencing has been employed to identify fungal pathogens. ITS regions have previously been used for the detection, identification, and classification of *Pyricularia* isolates [13]. A previous study confirmed that the ITS gene regions could identify *Pyricularia* at the species level, and sometimes lower than the species level [13,14]. In this study, we used the universal primers ITS5 and ITS4 to amplify the ITS region, because the ITS1 and ITS4 primer pair showed very poor success in our samples (the ITS1 and ITS4 primers can give rise to very poor amplification in some species [15]). Our results confirmed that all isolates used in this study belonged to the *P. oryzae* rice blast pathogen.

### 4.2. Genetic Diversity of Pyricularia oryzae Using SRAP and ISSR Markers

The analysis of genetic variation in plant pathogen populations is an important prerequisite for understanding coevolution in the plant pathosystem [16]. In the present study, based on ISSR and SRAP markers, SRAP markers produced a higher percentage of polymorphic bands (69.22%) than ISSR markers (52.90%). However, the average number of polymorphic bands from different markers detected by ISSR primers was 4.43, which was higher than that of SRAP primers (3.39). The dendrograms from the ISSR, SRAP, and ISSR+SRAP combined data showed slightly different groupings. We were unable to group the fungal strains when using ISSR data alone, while the SRAP dendrogram and PCA showed that most of the population tended to cluster together, with only 11 strains separated from the main group (10926, 10927, BKK55002, RBR55001, CCO56002, BKK55001, BKK55003, Guy11, 40.3, 70-15, and B1-2). Sequence-related amplified polymorphism (SRAP) markers amplify the ORF, so the dendrogram may relate to gene variation. The range of similarity in ISSR, SRAP, and ISSR+SRAP analyses were 0.27–1.00, 0.29–0.97, and 0.25–0.95, respectively. The fact that the similarity coefficients in different marker systems were comparable suggests that both markers were equally effective. Mixed multiple marker systems provide more complete genome information about both ORFs and microsatellites. Molecular phylogenetic grouping obtained by ISSR and SRAP analyses did not correlate with morphological characteristics, but morphology may affect conidia germination or pathogenicity. Ngernmuen et al. [17] developed SSR markers from whole genome sequences of rice blast fungus strains and evaluated the genetic diversity of rice blast strains from Thailand. The results showed a high level of genetic diversity, and blast strains could be clustered based on geographic distribution and collection time. Silva et al. [18] used rep-PCR analysis with two primer sequences from *Pot2* to assess genetic diversity of rice blast strains from Brazil. Xu et al. [19] used RAPD, REMAP, rep-PCR markers, and *AVR* gene sequences to examine the genetic diversity of rice blast fungus strains from China. The results revealed that the genetic structure of the *P. oryzae* population changed significantly over 3 years. Our results indicated that the rice blast population in Thailand does not explicitly group into different clades by site or year of sampling. The results from ISSR and SRAP data show low bootstrap values, suggesting clonal populations in our samples. The genetic variation supports the hypothesis that the main mechanism by which *P. oryzae* overcomes host resistant in natural conditions might be genome adaptation to avoid host recognition. Two main mechanisms for genome adaptation in fungi are sexual mating and parasexual recombination [20], but sexual reproduction in rice blast fungus has not been observed in the field and sexual spores have been produced only in laboratory settings. Therefore, parasexual recombination could most likely be the major mechanism for the exchange of DNA fragments [21] that causes genetic variation in the rice blast population. The genetic diversity and pathogen population structure from this study furthers our understanding of the rice blast fungus and could be useful for selecting rice cultivars or improving rice cultivars in the future.

## Figures and Tables

**Table 1:** Fungal morphological characteristics of 59 rice blast strains and their internal transcribed spacer (ITS) region.

Isolate	Origin ^(a)^	Year	Mycelium Features ^(b)^	Melanin Pigment ^(c)^	Conidia Shape ^(d)^	ITS ^(e)^
10100	Srakaew	2006	Cream, Fluffy, Densely	+	C	MT126183
10301	Sisaket	2006	White, Flat, Densely	++++	C	MT126184
10302	Sisaket	2006	White, Flat, Densely	+	B	MT126185
10459	Lampang	2006	Grey, Flat, Densely	++++	C	MT126186
10551	Nakhon Ratchasima	2006	White, Flat, Densely	++++	C	MT126187
10552	Nakhon Ratchasima	2006	White, Fluffy, Densely	+	C	MT126188
10576	Kalasin	2006	Cream, Fluffy, Densely	++++	C	MT126189
10577	Nakhon Ratchasima	2006	White, Flat, Densely	-	D	MT126190
10578	Nakhon Ratchasima	2006	Cream, Flat, Densely	+	C	MT126191
10581	Nakhon Ratchasima	2006	White, Fluffy, Densely	+++	C	MT126192
10652	Maha Sarakham	2006	Cream, Fluffy, Densely	+++	C	MT126193
10681	Roi Et	2006	White, Flat, Densely	++	B	MT126194
10694	Nong Khai	2006	Cream, Flat, Densely	+	B	MT126195
10732	Kamphaeng Phet	2006	White, Flat, Densely	-	C	MT126196
10760	Kamphaeng Phet	2006	White, Fluffy, Densely	-	D	MT126197
10812	Chiang Rai	2006	White, Flat, Densely	-	D	MT126198
10837	Surin	2006	White, Flat, Densely	++	B	MT126199
10873	Mae Hong Son	2006	Cream, Fluffy, Highly dense	++	C	MT126200
10926	Phayao	2006	White, Flat, Densely	-	C	MT126201
10927	Phayao	2006	White, Flat, Densely	-	C	MT126202
10941	Lampang	2006	White, Flat, Densely	-	C	MT126203
10971	Nan	2006	White, Flat, Densely	-	C	MT126204
10985	Sisaket	2006	Cream, Flat, Densely	+	C	MT126205
10993	Buriram	2006	White, Fluffy, Densely	+	C	MT126206
11100	Udon Thani	2006	White, Fluffy, Densely	+	C	MT126207
11108	Ubon Ratchathani	2006	White, Flat, Densely	++++	C	MT126208
11109	Ubon Ratchathani	2006	Cream, Fluffy, Densely	+	C	MT126209
BAG1.2	Phitsanulok	2006	White, Fluffy, Densely	+	C	MT126210
BAG4.6	Phitsanulok	2006	White, Flat, Densely	+++	D	MT126211
40.3	n/a	n/a	Grey, Fluffy, Densely	++	C	MT126212
SRN54001	Surin	2011	White, Flat, Densely	+++	D	MT126213
SRN54002	Surin	2011	White, Fluffy, Densely	++	D	MT126214
SRN54005	Surin	2011	White, Flat, Densely	++	D	MT126215
SRN54006	Surin	2011	White, Fluffy, Densely	++	C	MT126216
SRN54007	Surin	2011	Cream, Flat, Densely	++++	C	MT126217
SRN54009	Surin	2011	White, Flat, Densely	+++	C	MT126218
CCO56001	Chachoengsao	2013	Cream, Fluffy, Densely	++	C	MT126219
CCO56002	Chachoengsao	2013	White, Flat, Densely	++	C	MT126220
CCO56003	Chachoengsao	2013	Cream, Fluffy, Densely	++++	C	MT126221
CCO56004	Chachoengsao	2013	White, Fluffy, Densely	+	C	MT126222
CPM55001	Chaiyaphum	2012	Cream, Flat, Densely	+	C	MT126223
CPM55002	Chaiyaphum	2012	Grey, Flat, Densely	++++	C	MT126224
CPM55003	Chaiyaphum	2012	White, Fluffy, Densely	++++	C	MT126225
NYK55001	Nakhon Nayok	2011	Grey, Flat, Densely	++	C	MT126226
BKK55001	Bangkok	2011	White, Fluffy, Densely	+++	C	MT126227
BKK55002	Bangkok	2011	White, Flat, Densely	+	D	MT126228
BKK55003	Bangkok	2011	Grey, Fluffy, Densely	+++	C	MT126229
RBR55001	Ratchaburi	2011	Cream, Fluffy, Highly dense	++++	A	MT126230
RBR55002	Ratchaburi	2011	White, Flat, Densely	-	C	MT126231
RBR55003	Ratchaburi	2011	White, Fluffy, Densely	++	C	MT126232
B1-2	Ubon Ratchathani	2001	White, Fluffy, Densely	++++	C	MT126233
TH196031	Ubon Ratchathani	2001	White, Fluffy, Densely	++	C	MT126234
TH196036	Ubon Ratchathani	2001	White, Fluffy, Densely	++	C	MT126235
TRG1	Nong Khai	2013	Grey, Fluffy, Densely	+	C	MT126236
TRG2	Nong Khai	2013	White, Flat, Densely	-	C	MT126237
TRG3	Nong Khai	2013	Grey, Fluffy, Densely	++	C	MT126238
TRG4	Nong Khai	2013	Cream, Flat, Densely	-	C	MT126239
70-15	Laboratory strain	1988	Grey, Flat, Densely	-	C	MT126240
Guy11	French Guyana	1988	Grey, Fluffy, Densely	-	C	MT126241

^(a)^ Name of Thai province or the origin of blast sampling. ^(b)^ Fungal mycelia characteristics on rice flour agar (RFA), including color and mycelial appearance. ^(c)^ Melanin pigment production was observed at the ventral view of the culturing plate, scoring is as follows: No melanin pigment (-), melanin pigment production in less than 20% of the mycelia area (+), melanin production in 20%–50% of the mycelia area (++), melanin production in 50%–80% of the mycelia area (+++), and melanin production in more than 80% of the mycelia area (++++). ^(d)^ Conidial structure under light microscope. ^(e)^ GenBank accession number. A: Oval conidia, B: Short pyriform conidia, C: Pyriform conidia, and D: Long pyriform conidia.

**Table 2:** Polymorphism in 59 rice blast strains revealed by inter-simple sequence repeat (ISSR) primers.

Primer ID	Primer Name ^(a)^	Total Bands	Polymorphic Bands	% Polymorphic	PIC ^(b)^
ISSR1	UBC-807	12	4	33.33	0.39
ISSR2	UBC-808	14	8	57.14	0.15
ISSR3	UBC-809	8	2	25.00	0.49
ISSR4	UBC-811	8	3	37.50	0.16
ISSR5	UBC-812	11	4	36.36	0.49
ISSR6	UBC-813	3	3	100.0	0.49
ISSR7	UBC-814	0			
ISSR8	UBC-815	0			
ISSR9	UBC-817	6	2	33.33	0.26
ISSR10	UBC-818	5	1	20.00	0.38
ISSR11	UBC-820	0			
ISSR12	UBC-823	2	2	100.0	0.32
ISSR13	UBC-824	0			
ISSR14	UBC-825	8	4	50.00	0.50
ISSR15	UBC-826	10	9	90.00	0.50
ISSR16	UBC-827	0			
ISSR17	UBC-841	16	14	87.50	0.50
ISSR18	UBC-847	4	1	25.00	0.07
ISSR19	UBC-848	11	5	45.45	0.33
ISSR20	UBC-849	0			
	Total	118	62		
	Average	8.43	4.43	52.90	0.36

^(a)^ ISSR primers set from the University of British Columbia (Microsatellite UBC primer set 9, University of British Columbia, Vancouver, Canada). ^(b)^ The polymorphism information content (PIC) value was used to measure the informativeness of a genetic marker. Grey rows of the table denote primers that were unable to amplify the blast fungal genome.

**Table 3:** Polymorphism in 59 rice blast strains revealed by sequence-related amplified polymorphism (SRAP) primer combinations.

Primer ID	Primer Names ^(a)^	Total Bands	Polymorphic Bands	% Polymorphic	PIC ^(b)^
SRAP1	me1/em1	5	4	80.00	0.50
SRAP2	me1/em2	0			
SRAP3	me1/em3	5	5	100.0	0.49
SRAP4	me1/em4	0			
SRAP5	me1/em5	9	6	66.67	0.47
SRAP6	me1/em6	10	5	50.00	0.50
SRAP7	me2/em1	0			
SRAP8	me2/em2	11	4	36.36	0.50
SRAP9	me2/em3	0			
SRAP10	me2/em4	14	6	42.86	0.48
SRAP11	me2/em5	1	1	100.0	0.32
SRAP12	me2/em6	9	3	33.33	0.40
SRAP13	me3/em1	6	5	83.33	0.37
SRAP14	me3/em2	6	4	66.67	0.49
SRAP15	me3/em3	6	5	83.33	0.44
SRAP16	me3/em4	3	2	66.67	0.41
SRAP17	me3/em5	0			
SRAP18	me3/em6	6	4	66.67	0.38
SRAP19	me4/em1	2	2	100.0	0.39
SRAP20	me4/em2	5	1	20.00	0.03
SRAP21	me4/em3	0			
SRAP22	me4/em4	0			
SRAP23	me4/em5	0			
SRAP24	me4/em6	2	2	100.0	0.50
SRAP25	me5/em1	0			
SRAP26	me5/em2	0			
SRAP27	me5/em3	0			
SRAP28	me5/em4	1	1	100.0	0.34
SRAP29	me5/em5	2	1	50.00	0.03
SRAP30	me5/em6	0			
	Total	103	61		
	Average	5.72	3.39	69.22	0.39

^(a)^ SRAP markers were designed by Li and Quiros in 2001 to specifically amplify coding regions of genomes by targeting GC-rich exon regions (forward primers, named as me) and AT-rich intron regions (reverse primers, named as em). ^(b)^ The polymorphism information content (PIC) value was used to measure the informativeness of a genetic marker. Grey rows of the table denote primers that were unable to amplify the blast fungal genome.

## Supplementary Materials

The following are available online: https://www.mdpi.com/2309-608X/6/1/38/s1, Data S1: Fungal growth on rice flour agar (RFA) after 4, 6, 8, and 10 days after incubation; fungal mycelium were measured as the radius from the origin in millimeters. #1, 2, and 3 indicate individual replicates. #1 indicates replication 1, #2 indicates replication 2, and #3 indicates replication 3.
