# Supplementary material for: Morphological Characterization and Genetic Diversity of Rice Blast Fungus, Pyricularia oryzae, from Thailand Using ISSR and SRAP Markers
Source: J Fungi (Basel). 2020 Mar 19;6(1):38. doi: 10.3390/jof6010038 (PMC7151035; doi:10.3390/jof6010038)
Supplement: Supplementary file 1 [file jof-06-00038-s001.pdf]

**Supplement data 1**

| | 4 days | | | | | 6 days | | | | | 8 days | | | | | 10 days | | | | |
|---|---|---|---|---|---|---|---|---|---|---|---|---|---|---|---|---|---|---|---|---|
| isolate | #1 | #2 | #3 | Average | STDEV | #1 | #2 | #3 | Average | STDEV | #1 | #2 | #3 | Average | STDEV | #1 | #2 | #3 | Average | STDEV |
| 10100 | 10 | 9.5 | 9 | 9.50 | 0.50 | 16.5 | 17 | 16 | 16.50 | 0.50 | 23 | 23.5 | 23 | 23.17 | 0.29 | 28.5 | 29 | 28.5 | 28.67 | 0.29 |
| 10301 | 9.5 | 9 | 9 | 9.17 | 0.29 | 16 | 15.5 | 16 | 15.83 | 0.29 | 23.5 | 23 | 22.5 | 23.00 | 0.50 | 28 | 27.5 | 28 | 27.83 | 0.29 |
| 10302 | 7 | 8.5 | 8 | 7.83 | 0.76 | 13 | 15 | 15 | 14.33 | 1.15 | 20 | 20.5 | 21 | 20.50 | 0.50 | 24 | 24.5 | 25 | 24.50 | 0.50 |
| 10459 | 10.5 | 9 | 10 | 9.83 | 0.76 | 17.5 | 16 | 17 | 16.83 | 0.76 | 24 | 22.5 | 23 | 23.17 | 0.76 | 29 | 27.5 | 27 | 27.83 | 1.04 |
| 10551 | 9.5 | 9.5 | 11 | 9.83 | 0.58 | 16 | 16.5 | 16.5 | 16.33 | 0.29 | 23 | 23 | 24 | 23.33 | 0.58 | 26.5 | 27 | 28 | 27.17 | 0.76 |
| 10552 | 8 | 7.5 | 8.5 | 8.00 | 0.50 | 14 | 14 | 15 | 14.33 | 0.58 | 19.5 | 19 | 21 | 19.83 | 1.04 | 24 | 25 | 26 | 25.00 | 1.00 |
| 10576 | 8.5 | 10 | 9.5 | 9.33 | 0.76 | 16.5 | 17.5 | 17 | 17.00 | 0.50 | 23 | 24 | 24.5 | 23.83 | 0.76 | 30 | 30 | 30 | 30.00 | 0.00 |
| 10577 | 9.5 | 9 | 8.5 | 9.00 | 0.50 | 16 | 14 | 15.5 | 15.17 | 1.04 | 21 | 20 | 20.5 | 20.50 | 0.50 | 26 | 25 | 24.5 | 25.17 | 0.76 |
| 10578 | 8 | 8 | 8.5 | 8.17 | 0.29 | 13.5 | 14 | 14.5 | 14.00 | 0.50 | 18.5 | 21 | 20 | 19.83 | 1.26 | 24 | 26.5 | 26.5 | 25.67 | 1.44 |
| 10581 | 8 | 8.5 | 8 | 8.17 | 0.29 | 14.5 | 15 | 14.5 | 14.67 | 0.29 | 21 | 20.5 | 21.5 | 21.00 | 0.50 | 26 | 25.5 | 25.5 | 25.67 | 0.29 |
| 10652 | 10 | 9.5 | 9.5 | 9.67 | 0.29 | 17.5 | 16.5 | 15.5 | 16.50 | 1.00 | 24.5 | 25 | 23 | 24.17 | 1.04 | 30 | 30.5 | 29 | 29.83 | 0.76 |
| 10681 | 9.5 | 9 | 9.5 | 9.33 | 0.29 | 16 | 15.5 | 15.5 | 15.67 | 0.29 | 23 | 23 | 22 | 22.67 | 0.58 | 28 | 28 | 28 | 28.00 | 0.00 |
| 10694 | 8.5 | 9 | 9 | 8.83 | 0.29 | 15.5 | 6 | 14.5 | 12.00 | 5.22 | 21 | 21.5 | 21 | 21.17 | 0.29 | 25 | 25.5 | 25.5 | 25.33 | 0.29 |
| 10732 | 8 | 8.5 | 9 | 8.50 | 0.50 | 15 | 15 | 15.5 | 15.17 | 0.29 | 21 | 21.5 | 22 | 21.50 | 0.50 | 27 | 27 | 27 | 27.00 | 0.00 |
| 10760 | 8.5 | 9 | 8.5 | 8.67 | 0.29 | 14.5 | 15 | 14 | 14.50 | 0.50 | 21 | 21 | 21 | 21.00 | 0.00 | 27 | 27 | 28 | 27.33 | 0.58 |
| 10812 | 8.5 | 9 | 9 | 8.83 | 0.29 | 15 | 15 | 15 | 15.00 | 0.00 | 21 | 21 | 20 | 20.67 | 0.58 | 24.5 | 25 | 24.5 | 24.67 | 0.29 |
| 10837 | 7.5 | 8 | 9 | 8.17 | 0.76 | 14.5 | 15.5 | 15.5 | 15.17 | 0.58 | 20 | 19.5 | 22 | 20.50 | 1.32 | 24.5 | 23.5 | 25 | 24.33 | 0.76 |
| 10873 | 8 | 7 | 8 | 7.67 | 0.58 | 14.5 | 13 | 14.5 | 14.00 | 0.87 | 19 | 18 | 18.5 | 18.50 | 0.50 | 22 | 22 | 21.5 | 21.83 | 0.29 |
| 10926 | 8 | 8 | 9 | 8.33 | 0.58 | 14 | 14.5 | 14.5 | 14.33 | 0.29 | 19 | 20 | 20.5 | 19.83 | 0.76 | 24.5 | 25 | 26 | 25.17 | 0.76 |
| 10927 | 8 | 8 | 8 | 8.00 | 0.00 | 14 | 14 | 14.5 | 14.17 | 0.29 | 19.5 | 18.5 | 19 | 19.00 | 0.50 | 24 | 23.5 | 23.5 | 23.67 | 0.29 |
| 10941 | 8 | 8.5 | 8.5 | 8.33 | 0.29 | 15 | 15.5 | 15 | 15.17 | 0.29 | 20 | 21 | 20.5 | 20.50 | 0.50 | 26 | 27 | 26.5 | 26.50 | 0.50 |
| 10971 | 9 | 8.5 | 8 | 8.50 | 0.50 | 14.5 | 14.5 | 14 | 14.33 | 0.29 | 21 | 20.5 | 20 | 20.50 | 0.50 | 25.5 | 25.5 | 25 | 25.33 | 0.29 |
| 10985 | 8.5 | 8 | 9 | 8.50 | 0.50 | 15.5 | 15 | 16.5 | 15.67 | 0.76 | 21 | 20 | 22 | 21.00 | 1.00 | 25.5 | 25.5 | 27.5 | 26.17 | 1.15 |
| 10993 | 9 | 9 | 9 | 9.00 | 0.00 | 17 | 15.5 | 16 | 16.17 | 0.76 | 24.5 | 23.5 | 24 | 24.00 | 0.50 | 30 | 30 | 29.5 | 29.83 | 0.29 |
| 11100 | 8.5 | 8 | 8 | 8.17 | 0.29 | 14.5 | 14.5 | 15 | 14.67 | 0.29 | 22 | 21.5 | 22 | 21.83 | 0.29 | 27 | 26.5 | 27.5 | 27.00 | 0.50 |
| 11108 | 8.5 | 9.5 | 9.5 | 9.17 | 0.58 | 17 | 16.5 | 16.5 | 16.67 | 0.29 | 22 | 23.5 | 23 | 22.83 | 0.76 | 26.5 | 27 | 27.5 | 27.00 | 0.50 |
| 11109 | 9.5 | 9 | 9 | 9.17 | 0.29 | 15.5 | 15 | 15 | 15.17 | 0.29 | 20.5 | 20 | 21 | 20.50 | 0.50 | 25 | 25 | 25.5 | 25.17 | 0.29 |
| BAG1.2 | 8.5 | 9 | 9 | 8.83 | 0.29 | 16.5 | 15 | 15.5 | 15.67 | 0.76 | 23 | 24 | 23.5 | 23.50 | 0.50 | 28.5 | 30 | 29.5 | 29.33 | 0.76 |
| BAG4.6 | 9.5 | 9 | 9 | 9.17 | 0.29 | 15.5 | 15 | 16 | 15.50 | 0.50 | 23 | 23 | 23 | 23.00 | 0.00 | 29 | 29.5 | 30 | 29.50 | 0.50 |
| BKK55001 | 8.5 | 9 | 9 | 8.83 | 0.29 | 15.5 | 16 | 16.5 | 16.00 | 0.50 | 22 | 23 | 24 | 23.00 | 1.00 | 29 | 29 | 30 | 29.33 | 0.58 |
| BKK55002 | 8.5 | 8 | 8.5 | 8.33 | 0.29 | 13.5 | 13 | 14.5 | 13.67 | 0.76 | 20 | 20 | 20 | 20.00 | 0.00 | 24.5 | 24.5 | 24.5 | 24.50 | 0.00 |
| BKK55003 | 9.5 | 8.5 | 8 | 8.67 | 0.76 | 16 | 15.5 | 15 | 15.50 | 0.50 | 23 | 23.5 | 22.5 | 23.00 | 0.50 | 28.5 | 29 | 28 | 28.50 | 0.50 |
| CCO56001 | 9 | 8.5 | 8.5 | 8.67 | 0.29 | 16.5 | 16 | 17.5 | 16.67 | 0.76 | 23 | 21.5 | 23 | 22.50 | 0.87 | 28.5 | 27 | 28 | 27.83 | 0.76 |
| CCO55002 | 7.5 | 7.5 | 8.5 | 7.83 | 0.58 | 15.5 | 15.5 | 16 | 15.67 | 0.29 | 23 | 22.5 | 23 | 22.83 | 0.29 | 27.5 | 28 | 29.5 | 28.33 | 1.04 |
| CCO56003 | 10 | 10 | 10 | 10.00 | 0.00 | 17.5 | 17 | 16.5 | 17.00 | 0.50 | 24 | 23.5 | 24 | 23.83 | 0.29 | 30 | 29 | 29 | 29.33 | 0.58 |
| CCO56004 | 8 | 8.5 | 9 | 8.50 | 0.50 | 16 | 15.5 | 16.5 | 16.00 | 0.50 | 23 | 23 | 22.5 | 22.83 | 0.29 | 27.5 | 28 | 28 | 27.83 | 0.29 |
| CPM55001 | 8 | 8 | 8.5 | 8.17 | 0.29 | 15 | 14.5 | 15 | 14.83 | 0.29 | 19 | 19.5 | 19.5 | 19.33 | 0.29 | 24 | 24 | 24.5 | 24.17 | 0.29 |
| CPM55002 | 9 | 9 | 9 | 9.00 | 0.00 | 16.5 | 16.5 | 16.5 | 16.50 | 0.00 | 23.5 | 23 | 23.5 | 23.33 | 0.29 | 29 | 28.5 | 29 | 28.83 | 0.29 |
| CPM55003 | 9 | 9.5 | 9.5 | 9.33 | 0.29 | 16 | 15.5 | 16 | 15.83 | 0.29 | 24 | 23.5 | 23.5 | 23.67 | 0.29 | 29 | 29 | 29.5 | 29.17 | 0.29 |
| NYK55001 | 8 | 8 | 8.5 | 8.17 | 0.29 | 14.5 | 13.5 | 15 | 14.33 | 0.76 | 20 | 20 | 20 | 20.00 | 0.00 | 30 | 30 | 29.5 | 29.83 | 0.29 |
| RBR55001 | 8 | 10 | 9.5 | 9.17 | 1.04 | 14 | 15 | 15 | 14.67 | 0.58 | 18 | 18 | 18.5 | 18.17 | 0.29 | 20 | 22 | 21.5 | 21.17 | 1.04 |
| RBR55002 | 7.5 | 7.5 | 7 | 7.33 | 0.29 | 13 | 12.5 | 13 | 12.83 | 0.29 | 17.5 | 18 | 18 | 17.83 | 0.29 | 21.5 | 22 | 21.5 | 21.67 | 0.29 |
| RBR55003 | 8.5 | 9 | 9 | 8.83 | 0.29 | 14 | 15 | 15 | 14.67 | 0.58 | 21.5 | 23 | 22.5 | 22.33 | 0.76 | 28.5 | 27.5 | 27.5 | 27.83 | 0.58 |
| SRN54001 | 10.5 | 9.5 | 9 | 9.67 | 0.76 | 17 | 16.5 | 14.5 | 16.00 | 1.32 | 24 | 23 | 22 | 23.00 | 1.00 | 28.5 | 28.5 | 27 | 28.00 | 0.87 |
| SRN54002 | 8.5 | 8.5 | 8 | 8.33 | 0.29 | 15.5 | 15.5 | 15 | 15.33 | 0.29 | 22.5 | 20 | 22 | 21.50 | 1.32 | 28 | 26 | 27 | 27.00 | 1.00 |
| SRN54005 | 9 | 9.5 | 9 | 9.17 | 0.29 | 15.5 | 16 | 15.5 | 15.67 | 0.29 | 24 | 23 | 22.5 | 23.17 | 0.76 | 29.5 | 29 | 29 | 29.17 | 0.29 |
| SRN54006 | 10 | 10 | 8.5 | 9.50 | 0.87 | 16 | 16.5 | 17 | 16.50 | 0.50 | 23.5 | 23 | 24 | 23.50 | 0.50 | 30 | 28.5 | 29.5 | 29.33 | 0.76 |
| SRN54007 | 9 | 9 | 8.5 | 8.83 | 0.60 | 15 | 17 | 16 | 16.00 | 1.00 | 23 | 23 | 23 | 23.00 | 0.00 | 29 | 29 | 29 | 29.00 | 0.00 |
| SRN54009 | 7.5 | 8.5 | 9.5 | 8.50 | 1.00 | 13.5 | 14.5 | 15 | 14.33 | 0.76 | 19.5 | 20 | 20.5 | 20.00 | 0.50 | 24 | 25 | 26 | 25.00 | 1.00 |
| 40.3 | 10 | 9.5 | 8.5 | 9.33 | 0.76 | 17.5 | 17.5 | 16.5 | 17.17 | 0.58 | 25 | 25 | 24 | 24.67 | 0.58 | 30 | 30.5 | 30.5 | 30.33 | 0.29 |
| B1-2 | 9.5 | 9.5 | 9 | 9.33 | 0.29 | 16.5 | 16 | 16.5 | 16.33 | 0.29 | 22.5 | 22 | 23 | 22.50 | 0.50 | 27.5 | 28 | 28 | 27.83 | 0.29 |
| TH196031 | 10 | 9.5 | 10 | 9.83 | 0.29 | 16 | 16 | 18 | 16.67 | 1.15 | 23.5 | 23 | 25 | 23.83 | 1.04 | 28 | 28 | 28.5 | 28.17 | 0.29 |
| TH196036 | 9.5 | 10 | 9 | 9.50 | 0.50 | 17 | 17.5 | 16.5 | 17.00 | 0.50 | 23.5 | 24 | 24 | 23.83 | 0.29 | 29 | 29.5 | 29.5 | 29.33 | 0.29 |
| TRG1 | 8.5 | 9 | 8.5 | 8.67 | 0.29 | 14 | 16.5 | 16 | 15.50 | 1.32 | 22 | 23 | 23.5 | 22.83 | 0.76 | 29 | 29 | 29 | 29.00 | 0.00 |
| TRG2 | 9 | 8.5 | 8 | 8.50 | 0.50 | 15 | 14 | 14 | 14.33 | 0.58 | 19.5 | 19 | 19 | 19.17 | 0.29 | 23 | 23 | 22.5 | 22.83 | 0.29 |
| TRG3 | 9 | 9.5 | 9 | 9.17 | 0.29 | 17 | 17 | 17 | 17.00 | 0.00 | 23.5 | 24 | 23.5 | 23.67 | 0.29 | 29 | 28.5 | 29 | 28.83 | 0.29 |
| TRG4 | 9 | 8.5 | 9 | 8.83 | 0.29 | 16 | 14.5 | 15.5 | 15.33 | 0.76 | 20 | 20 | 20.5 | 20.17 | 0.29 | 23.5 | 23.5 | 23 | 23.33 | 0.29 |
| 70-15 | 8 | 8 | 8.5 | 8.17 | 0.29 | 13.5 | 12 | 13.5 | 13.00 | 0.87 | 19 | 19.5 | 20 | 19.50 | 0.50 | 24 | 25 | 25 | 24.67 | 0.58 |
| Guy11 | 9 | 8 | 10 | 9.00 | 1.00 | 15.5 | 16 | 15.5 | 15.67 | 0.29 | 24 | 23.5 | 22 | 23.17 | 1.04 | 29 | 29.5 | 28 | 28.83 | 0.76 |
| | | | Max | 10.00 | | | | Max | 17.17 | | | | Max | 24.67 | | | | Max | 30.33 | |
| | | | Min | 7.33 | | | | Min | 12.00 | | | | Min | 17.83 | | | | Min | 21.17 | |
| | | | Avr | 8.79 | | | | Avr | 15.36 | | | | Avr | 21.78 | | | | Avr | 26.94 | |
| | | | Std | 0.60 | | | | Std | 1.13 | | | | Std | 1.76 | | | | Std | 2.34 | |
